# Understanding the Current Distribution and Mass Transport Properties in 3D-Printed Architected Flow-Through Electrodes

**DOI:** 10.1021/acsaenm.4c00561

**Published:** 2025-01-17

**Authors:** Auston L. Clemens, Kyle Jung, Massimiliano Ferrucci, Megan E. Ellis, Jonathan T. Davis, Swetha Chandrasekaran, Zhen Qi, Christine A. Orme, Marcus A. Worsley, Rohan Akolkar, Anna Ivanovskaya, Nikola A. Dudukovic

**Affiliations:** † Materials Engineering Division, Engineering Directorate, Lawrence Livermore National Laboratory, Livermore, California 94550, United States; ‡ Materials Science Division, Physical and Life Sciences Directorate, Lawrence Livermore National Laboratory, Livermore, California 94550, United States; § Department of Chemical and Biomolecular Engineering, Case Western Reserve University, Cleveland, Ohio 44106, United States

**Keywords:** electrodeposition, additive manufacturing, lattice, electrode, functional coating, Wagner number

## Abstract

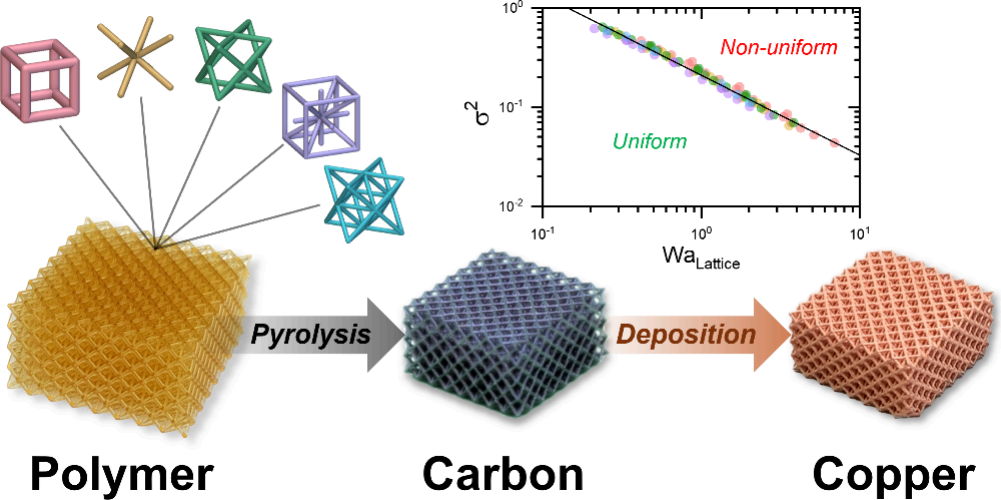

Architected materials offer promising advancements in energy storage by enabling highly customizable, high-surface-area, ordered, and low-defect porous structures. This study investigates the current distribution and mass transport within complex 3D-printed lattice electrodes under flow-through conditions. Conductive lattices were fabricated using microstereolithography followed by pyrolytic carbonization. Lattice geometry effects were analyzed by varying the unit cell type [simple cubic (SC), body- and face-centered cubic (BCC/FCC), IsoTruss, and Octet], porosity, and current density. Current distribution uniformity was investigated using a model high-efficiency copper deposition reaction. Local film thickness distributions were predicted using a numerical model and validated experimentally using micro-X-ray computed tomography. Scaling relationships for informing electrochemical reaction conditions and current uniformity are formulated as a modified lattice-based Wagner number (*Wa*_Lattice_) and a corresponding inverse Damkohler number (*Da*_Lattice_^–1^). Validated models reveal that mass-transfer coefficients scale as Octet > IsoTruss > FCC ∼ BCC > SC. Inertial effects become significant at Reynolds number *Re* > 3 and are particularly pronounced in Octet structures due to an abundance of struts oriented away from the fluid flow direction. The study underscores the importance of electrode engineering and process conditions necessary to tailor mass transport and current uniformities to various device applications.

## 1 Introduction

In the swiftly evolving global landscape characterized by escalating energy demands and pressing environmental concerns, a critical need arises for substantial strides in material development within energy storage and conversion devices. This urgency is particularly pronounced in redox flow batteries, water electrolyzers, and fuel cells, as pivotal devices that address off-hour energy demands and portable energy solutions.^1−3^ Among the various factors that influence the functionality of these devices, the geometric design of electrodes can play a significant role in determining their overall effectiveness.^4−9^ Traditional planar and/or carbon felt or woven electrodes often struggle to achieve both high current densities and uniform distribution of currents; however, electrodes that have a periodic structure tend to perform better as a result of improved fluid transport.^10−13^ Commercially manufactured felt electrodes feature a wide distribution of pore sizes which can manifest into issues associated with hotspots, or regions of localized electrolyte depletion due to fluid channeling which can hinder their performance.

To address these challenges, the use of 3D-printed architected materials as ordered lattice electrodes has emerged as a promising solution.^14^ Architected electrodes bear several advantages compared to traditional planar electrodes across various electrochemical applications. These 3D beam-based porous networks provide expanded surface areas for surface reactions to occur, yielding augmented reaction rates and heightened efficiency. Operationally, flow-through electrodes eliminate the need for flow fields that attempt to distribute electrolyte evenly across the electrode surface, which often still results in significant concentration gradients.^15,16^ Additionally, the intricate structures facilitate superior mass transport of reactants and products under flow,^14,17−19^ countering diffusion limitations, and disrupting development of large boundary layers that can plague conventional planar electrodes.^10,20,21^ Additive manufacturing allows the fabrication of tailored electrode designs that have been optimized for various reactions and operational conditions,^15,22^ a level of adaptability not easily attainable with conventional planar electrodes. Some examples include designing electrodes for the efficient release of evolved gas bubbles at hydrogen evolution reaction electrodes.^23−25^ Electrodes can also be designed to leverage the effects of surface tension to design preferentially wetted electrode structures for complex control of electrolyte-electrode interfaces.^26^ Examples of architected electrodes can be found in supercapacitors,^27,28^ batteries,^29^ water and CO_2_ electrolysis^23−25,30^ and biosensing devices.^31−33^

Architected materials are typically fabricated through additive manufacturing techniques such as powder bed fusion (PBF), direct ink writing (DIW), and stereolithography (SLA), to name a few. For fabrication of conductive electrodes, each technique possesses advantages and disadvantages. PBF can be used to generate metal structures and customizable electrodes over a wide range of devices;^34,35^ however, it can be limited in its feedstock flexibility, cost, and print resolution. DIW can be used to print slurries that can subsequently be processed to produce conductive metallic or carbon structures for electrochemical applications such as capacitors,^36,37^ water electrolysis,^38^ and vanadium redox flow batteries;^22^ it is however limited in achievable geometric complexity and resolution. SLA printing has advantages of high resolution (tens of micrometers) and the ability to produce parts with large overhangs. While the resins—typically acrylate polymers—are not inherently conductive, the printed part can be judiciously annealed and pyrolyzed into detailed yet conductive carbon scaffolding.^39^ Carbon lattices produced in this manner are lightweight and maintain good compressive strength,^40,41^ making them well-suited for energy storage solutions.

Current nonuniformity within 3D electrodes remains a significant challenge inherently characteristic of energy storage devices, stemming from the interplay of geometric complexity and mass transport limitations.^8^ Improving print resolution can yield electrodes with smaller unit cells and higher total surface areas for electrochemical reaction, but conversely confine more nonuniform electric fields and produce higher pressure drops for facilitated mass transport. Therefore, the optimal unit cell or feature size will depend on the end use application. Hence, there is a need for quantifiable metrics to compare electrode performance across different architected electrode unit cell types and sizes. Many studies infer mass transport behavior within 3D flow-through electrodes by conducting mass transport analysis at limiting current conditions. Dimensionless parameters are used to identify optimal operating conditions and/or electrode geometries to maximize reaction rate and minimize pressure drop within the device independent of macroscale cell geometry.^42−44^ These experiments, although helpful, do not accurately describe current distribution (or potential distribution) under mixed kinetic and mass transport control conditions, nor do they identify features or regions that develop inhomogeneous concentration profiles at the electrode when insufficient fluid flow is present. The durability and performance of devices that utilize plating processes such iron redox flow batteries (RFBs) and rechargeable batteries are highly affected by these nonuniform current distributions due to inhomogeneous metal deposits and dendrite formation.^45^ Within CO_2_ electrolysis, 3D flow through electrodes can increase reaction rates and single pass CO_2_ conversion.^6^ However, the reduction product distribution is highly sensitive to differences in local overpotentials.^46^ Understanding and mitigating these nonuniformities are crucial for optimizing the overall performance and durability of advanced energy storage systems.

**Figure 1:**
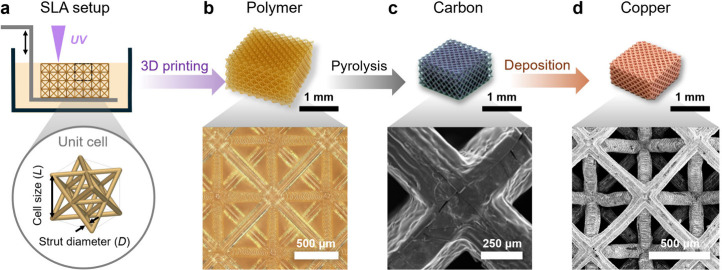
Design and fabrication of architected electrodes. (a) Schematic of the SLA setup for 3D printing of architected lattices and a representative octet unit cell with pertinent design features: cell size (*L*) and strut diameter (*D*). (b) Printed polymer octet lattice. (c) Pyrolyzed carbon octet lattice. (d) Copper-electroplated octet electrode.

Here, our study seeks to develop design rules and operating conditions for achieving uniform current distribution in architected electrodes. Using SLA printing and pyrolysis, we fabricate carbon lattices of varying unit cells and densities as substrates (Figure 1). We focus on electrodeposition of copper as a model reaction. We use micro-X-ray computed tomography (μCT) to visualize structural changes in the electrode, similarly done in batteries,^43,45^ commercial fiber electrodes,^47,48^ and in RFBs.^49^ The thickness of the deposited copper was measured and used as a proxy for local current density under varying deposition rates. Computational fluid dynamics modeling complements these insights, contributing to a comprehensive understanding of structured electrode behavior under fluid flow conditions. Finally, we discuss approaches to scaling analysis and introduce a modified Wagner number (*Wa*_Lattice_) offering predictive insights into macroscopic coating uniformity and thus current distribution as well as a modified Damköhler number (*Da*_Lattice_^–1^) to infer mass-transfer effects of the unit cell geometry on submicron level nonuniformities or defects. We find that specific unit cell architectures facilitate more tortuous fluid mixing at higher flow rates whereas others are more susceptible to boundary layer effects regarding orientation relative to the direction of flow which manifest in differences in mass transport engineering correlations. This exploration sets the stage for advancing the performance and applicability of energy storage and conversion devices through advanced 3D electrode designs, shedding light on a critical aspect of their functionality.

## 2 Results and Discussion

### 2.1 Fabrication of Architected Carbon Electrodes

**Figure 2:**
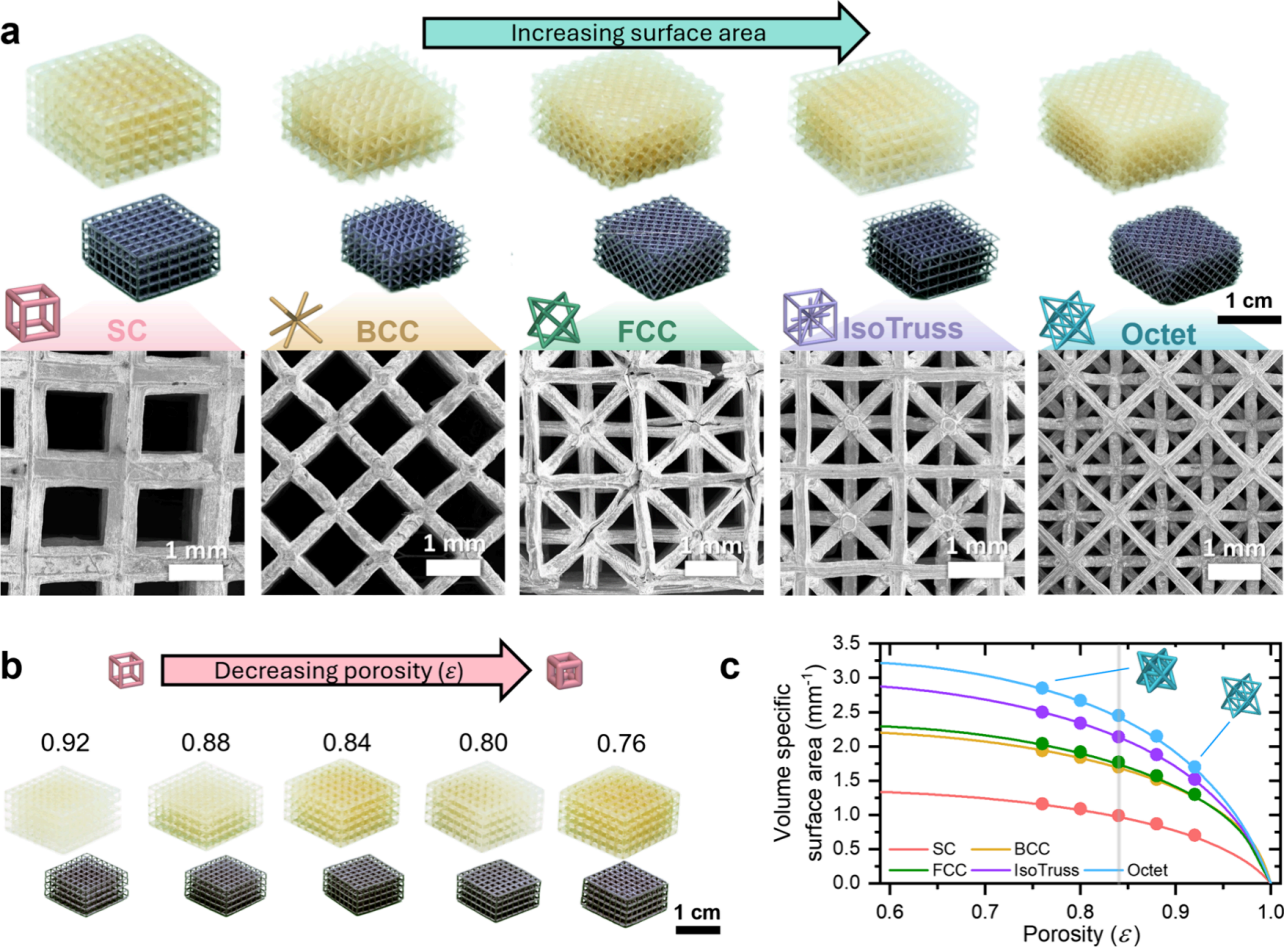
Varying lattice architecture across a range of unit cells and porosities. (a) Photographs of printed and carbonized lattices (top) and SEM images of copper-coated lattices (bottom) of different unit cells at the same porosity (ε = 0.84). From left: SC, BCC, FCC, IsoTruss, and Octet. (b) SC lattices of varying porosities. (c) Volumetric specific surface area of modeled geometries and corresponding cell architectures. The gray line denotes the sample porosity ε = 0.84 corresponding to images shown in part a.

Architected beam-based lattice ordered electrodes were fabricated by using SLA, as demonstrated by Figure 1. A commercial acrylate-based resin (Prototyping Resin PR48 Clear, Arkema USA) was used to print detailed, high resolution, electrodes using a custom microstereolithography 3D printer.^50^ The PR48 resin responds favorably to pyrolysis treatment due to its highly cross-linked network. A series of beam-based unit cell architectures were designed and fabricated to probe how geometry affects current uniformity in architected electrodes. To study the effect of lattice architecture, five different unit cell types were chosen: simple cubic (SC), body-centered cubic (BCC), face-centered cubic (FCC), IsoTruss, and Octet (Figure 2a). Each unit cell features varying amounts of intersecting beams, geometric variations, and resulting geometrical surface area. To study the effects of lattice density, the porosity (ε) of the lattice structures was varied from 0.92 to 0.76 (Figure 2b).

The printed parts were thermally postcured at 300 °C to minimize warping and shrinkage, and subsequent pyrolysis was carried out with a gradual temperature ramp. At elevated temperatures (>500 °C), the well-formed cross-linked thermoset network evolves into carbon scaffold amenable to conducting current while subsequently holding structure in place.^29^ Additional carbon structuring occurs at temperatures below 800 °C. The processing condition of 1000 °C was chosen to maximize conductivity and mechanical integrity.^30^ While macroscopic warping was minimal, microvoids and cracks can still occur as pyrolysis gases escape (Figure S1). Printed struts below 100 μm in diameter are susceptible to significant warping during heat treatment, resulting in poor handleability of the electrodes. For this reason, porosity ε of the samples were varied from 0.76 to 0.92 across all cell types for this study to span a significant range of volumetric specific surface areas (Figure 2c). Further decrease in porosity ε < 0.76 results in diminishing return in surface area.^51^ Octet and IsoTruss structures feature significantly higher surface areas over conventional SC structures due to the increase amount of slimmer intersecting struts. At the highest studied porosity, the strut diameter of the Octet structure with *L* = 1.75 mm is *D* = 211 μm (Table 1), providing sufficient margin for error and mechanical integrity to allow for compression during electroplating.

**Table 1:** Table of Geometric Design Parameters of Tested Architected Electrodes

unit cell type (ε = 0.84)	volume specific surface area (cm^–1^)	printed relative strut size (*D*/*L*)	carbonized relative strut size (*D*/*L*)	carbonized unit cell size (mm)
SC	15.91	0.286	0.276 ± 0.009	1.72 ± 0.016
BCC	17.70	0.188	0.163 ± 0.009	1.73 ± 0.031
FCC	22.51	0.170	0.157 ± 0.004	1.94 ± 0.050
IsoTruss	25.87	0.137	0.145 ± 0.004	1.72 ± 0.024
Octet	29.18	0.121	0.134 ± 0.006	1.75 ± 0.016

The final structure of the printed electrodes shrinks to ∼70% of its original volume and ∼90% of its original mass. All tested architectures resulted in final carbonized parts at 70% of the printed size within 5% except for FCC structures where the strut diameters were 15% larger, which can be attributed to the cracking that occurs at intersection points that allow for a larger final product than would otherwise. Despite these discrepancies, the manufactured structures maintain their shape, amenable for comparison to idealized CAD representations for numerical modeling. The carbonization process yielded electrodes with an average conductivity of ∼500 S m^–1^. The finalized structures are then modeled accordingly. The resulting structure yields a smooth, hydrophobic, and often texture-less surface (Figure S2), susceptible to entrapping bubbles generated during the electrodeposition process. The low surface roughness also makes nucleation onto carbon electrodes difficult. To mitigate these issues, the lattices were treated with oxygen plasma for 1 h. The final carbon electrode features increased surface roughness and improved wetting with aqueous solutions (Figure S3) as a result of surface oxidation and desorption of CO_2_ byproducts.^52,53^

### 2.2 Current Uniformity Modeling

#### 2.2.1 Architected Electrode Scaling Analysis

Electrodeposition was conducted on the various carbon architected electrodes as flow-through electrodes housed inside a custom printed flow cell. Current densities ranging from 0.35 to 1.4 mA cm^–2^ were used to produce smooth electrodeposited films suitable for coating thickness analysis. Conventional scaling parameters can be calculated for simple geometries such as cylindrical flow-through electrodes to infer relative nonuniformities of current density upon the electrode surface.^54,55^ Deriving dimensionless groups for nonstandard geometries such as unit cell based architected structures can be challenging. Therefore, geometric averaging across the relevant length scales is used to generate the equivalent Wagner number (*Wa*) for architected materials. *Wa* represents the ratio of the resistance to activation (*R*_a_), as determined by Tafel kinetics, to the ohmic resistance (*R*_Ω_) across the electrolyte. *Wa* is used as a tool to predict the current distribution of a system based on the geometry and process conditions.

**Figure 3:**
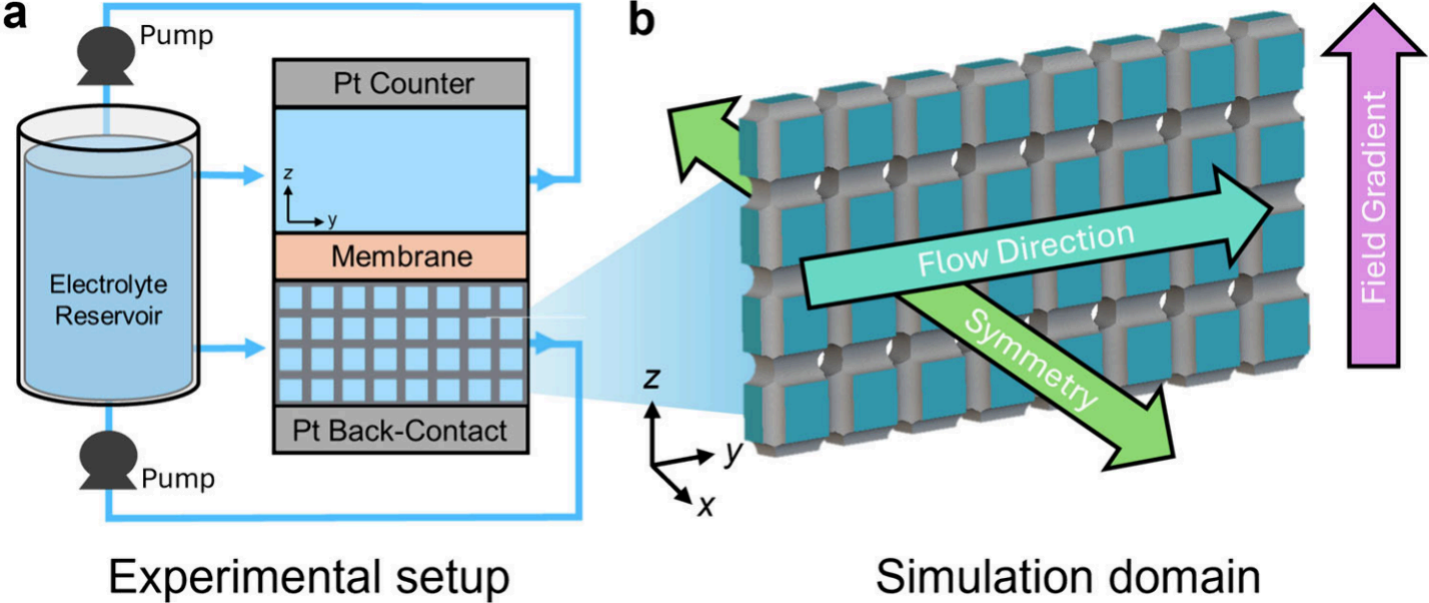
Electrodeposition of copper onto carbonized lattices. (a) Schematic illustration of the custom flow cell reactor. (b) CAD geometry of the fluid domain (blue) used in the numerical simulation. Arrows indicate general simulation constraints. The gray domain indicates the lattice surface where electrodeposition occurs.

The architected electrodes in this study were constrained between a platinum film back contact and a proton exchange membrane that sandwiched the electrodes into place, as shown in Figure 3a, similar to flow-battery-type configurations. A corresponding platinum current collector is used on the backside of the 3D electrode. Here, the total surface area (*A*_s_) of the 8 × 8 × 4 unit cell electrode is given in eq 1 as the number of unit cells perpendicular to the electric field (*N*_*z*_) multiplied by the surface area per unit cell (*S*_u_):
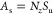
1where *N*_*z*_ = 4 in this example. *R*_a_ is then rederived as the derivative of the activation overpotential with respect to the current, calculated at the average current density (eq 2). With the prior geometric definitions and assuming Tafel kinetics, we get
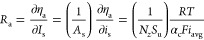
2where η_a_ is the activation overpotential, *I*_s_ is the total reaction current at the electrode, *i*_s_ is the local surface current density, *R* is the ideal gas constant, *T* is the temperature, α_c_ is the cathodic charge-transfer coefficient, *F* is Faraday’s constant, and *i*_avg_ is the average current density of the electrode. Additionally, the ohmic resistance *R*_Ω_ can be calculated assuming Ohm’s law applied to the ionic current flowing across an average cross section of fluid electrolyte. The cross section of the electrode perpendicular to flow is also approximated assuming an average cross section defined by the porosity of the sample (ε), as given in eq 3:
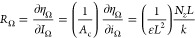
3where η_Ω_ is the ohmic overpotential, *I*_Ω_ is the total current through the electrolyte, *A*_c_ is the cross-sectional area in the *z* direction, *i*_Ω_ is the local current density through the electrolyte, *L* is the size of the unit cell, and *k* is the conductivity of the electrolyte. The final modified dimensionless parameter for determining the relative current uniformity within architected materials *Wa*_Lattice_ becomes (eq 4):
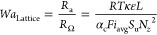
4

Additional scaling parameters such as the Damköhler number (*Da*), presented here as the inverse Damköhler number (*Da*_Lattice_^–1^) given in eq 5, describes the rate of reaction relative to rate of electroactive species transport:^56^
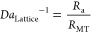
5where *R*_MT_ is the resistance to mass transport (eq 6), which accounts for diffusional and convective resistances in parallel:
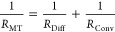
6

At the investigated flow rates, diffusion effects are negligible compared to convection, giving^55^
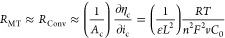
7Equations 6 and 7 then result in the following definition of *Da*_Lattice_^–1^ for architected electrodes (eq 8):
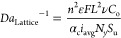
8where *n* is the number of electrons, ν is the average velocity, *C*_o_ is the concentration of electroactive species, and *N*_*y*_ is the number of unit cells in the direction of flow.

#### 2.2.2 Architected Electrode Numerical Modeling

Models of the fluid domain were generated for each unit cell type including the half-cell geometry assuming symmetrical behavior about the center of each cell. A representative 1 × 8 × 4 row of cells was used assuming minimal variation in flow field variation within the *xz* plane, as depicted in Figure 3b. The model geometry includes the unit cell centers and thus does not capture the outer half of all exterior struts. Specific geometric values of each unit cell type modeled are represented in Table 1. Numerical simulations were carried out for all unit cell types and porosities shown in Figure 2.

Under adequate transport of electroactive species (*Da*_Lattice_^–1^ → ∞) we can expect minimal concentration profiles to develop at the electrode surfaces. However, for periodic materials with changing ratios of surface area and cross sections in the direction of flow, submicron level regions of concentration-dependent inhomogeneities can develop. The behavior of the architected electrode current distribution is largely dependent on the geometric ability to facilitate mixing and avoid the development of significant boundary layer effects. For this reason, *C*_o_ = 10 mM was chosen to capture the concentration dependent effects at low current densities that otherwise would not manifest under sufficiently high concentration of electrolyte.

### 2.3 μCT and Validation

#### 2.3.1 Validation of Macroscopic Uniformity

**Figure 4:**
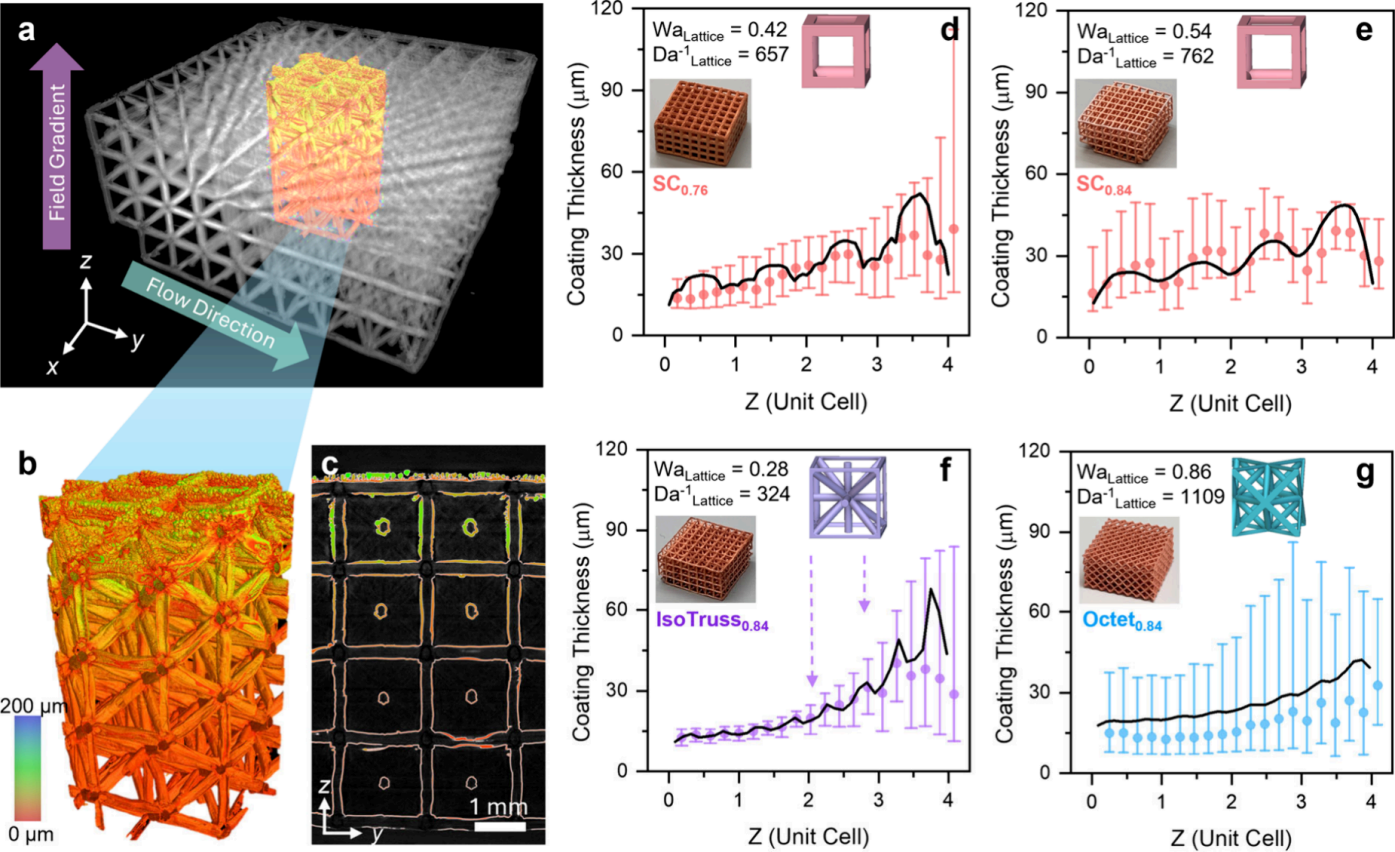
Analysis of electrodeposited copper coatings using μCT. (a) μCT reconstruction of an entire coated IsoTruss electrode (1× objective lens and 10 μm voxel size) highlighting the ROI scanned at higher resolution (4× objective lens and 2.5 μm voxel size). (b) 3D reconstruction of ROI used for data processing. (c) Exemplary *xz* plane of the ROI IsoTruss sample. (d–g) Plots of the average coating thickness as a function of distance (scaled by unit cell size) from the back contact. A higher *Z* indicates closer to the counter electrode. Measured μCT analysis of electrodeposited coating thickness (symbols) and numerical simulation data at experimental conditions (black line). Error bars represent the 5th and 95th percentile of the measured distribution of coating thicknesses using the ray method. Each data point represents a sample region of 0.35 mm in the *z* direction. Corresponding dimensionless parameters at experimental conditions are provided.

Several representative electroplated electrodes were measured using μCT to validate numerical model data of the electrodeposited film thickness and predict uniformity. To obtain sufficient signal-to-noise ratio, a region of interest (ROI) about the center of the architected electrode was chosen for higher resolution sampling, as shown in Figure 4a,b. A 4× objective lens was used to obtain a cylindrical ROI of 5 mm diameter and 2.5 μm voxel size. Galvanostatic electrodeposition was conducted for up to 64 h to generate a coating with an average thickness of 27.5 μm, >10× that of our pixel resolution. The measured coating thickness was resolved using VGStudio software, using the ray tracing method.^57^

SC structures with different porosities were chosen to highlight the effect of porosity on current distribution. In SC_0.76_, the numerical prediction provided Figure 4d lies within the error of the μCT measurements except for the topmost surface (*Z* ∼ 4). The average coating thickness is likely higher than μCT measurements suggest due to the uneven morphology and difficulty in approximating these irregular boundaries using the ray tracing method. A few additional defects are found in this sample which may have contributed to slight differences in prediction and our measurement. Within the less dense SC_0.84_, the electrodeposited coating featured a more optically and topographically smooth and defect-free coatings and resulted in excellent agreement with numerical predictions (Figure 4e). A minimal difference in macroscopic uniformity was evident between the two samples due to the relatively small difference in *Wa*_Lattice_ from 0.42 to 0.54. Additionally, a representative IsoTruss_0.84_ sample was electroplated at the same average current density to assess variance in structure complexity. Similarly to SC, increases in coating thickness significantly increases as *Z* approaches the counter electrode facing edge. For both SC_0.76_ and IsoTruss_0.84_, the topmost coatings are rough, which resulted in underprediction and large error bars. In IsoTruss_0.84_, the rough topmost coating is visually thicker than measured via μCT which would indicate a more nonuniform coating than the SC structures which agrees with the smaller *Wa*_Lattice_ value. Coating thickness measurements below *Z* < 3.5 are normally and narrowly distributed due to the well behaved and featureless copper deposits throughout the remainder of the samples and agree well with simulations.

To demonstrate the ability to achieve uniform current distribution behavior, the Octet_0.84_ lattice was chosen for deposition at a lower current density (0.35 mA cm^–2^) at *Wa*_Lattice_ = 0.86 for 64 h to generate a 27.5 μm nominal coating thickness. In this demonstration, the plated lattice yielded rough deposits, likely resulting from nucleation effects present at lower overpotentials, similarly to those observed on simpler carbon-based flow through electrodes.^54^ In future efforts, coating quality can be improved by implementing a two-step approach with differing current densities. An initial short period of increased current density would drive higher nucleation density and more uniform distribution of nuclei. The subsequent step would proceed as prolonged deposition at a lower current density, required for achieving uniform film growth.^58^ The distribution of measurements, due to the rough deposits, are wider and skewed by region of thicker deposits however over the entire ROI sampling, the current density trend follows the numerical predictions. Despite roughness considerations, the distribution of current throughout the Octet_0.84_ sample is much more uniform than other samples studied. This can be attributed to the higher *Wa*_Lattice_ of 0.86 relative to 0.24 for IsoTruss_0.84_. Despite the additional geometric complexity, it is shown that the Octet_0.84_ sample can be plated more uniformly by reducing the current density by 1/4 compared to IsoTruss_0.84_. The coating at the topmost surface measured an average coating thickness of ∼30 μm as opposed to 15 μm at the bottom most surface. This 2× difference represents a significant improvement over the IsoTruss sample that represents a conservative 4.6× difference end-to-end on account of the error associated with the ray tracing method. By leveraging the *Wa*_Lattice_ scaling relationship, we can predict the relative macroscopic current distribution uniformity; however, additional considerations are needed to account for concentration effects to more effectively use the scaling relationship provided here.

#### 2.3.2 Concentration-Dependent Effects

The μCT data can resolve submicron regions of inhomogeneous deposition due to local depletion of the copper species and reflected by the reduction in the macroscopic measure of the root-mean-square uniformity. Differences between upstream and downstream facing surfaces of vertical struts in the SC and IsoTruss structures can be measured. In agreement with simulation, the top unit cell (*Z* = 3–4) upstream-facing surfaces measured a ∼60% thicker deposit compared to downstream surfaces, as exemplified by the *xz* plane of IsoTruss_0.84_ presented in Figure 4c. Specific features such as the nodes of the lattice show signs of concentration depletion. This is evident by dendrite-like growth rooted at intersecting points at top of the structure, characteristic of deposit growths as they approach mass transport limiting current.^45,46^ Extrapolation near rate-limiting current over 16 h resulted in large nodules that formed at these points. Other points in which these phenomena occur are at the topmost surface that lies in contact with the proton exchange membrane providing mechanical compression. Complete μCT analysis of the representative copper plated electrodes studied here are presented in Figures S5–S8.

**Figure 5:**
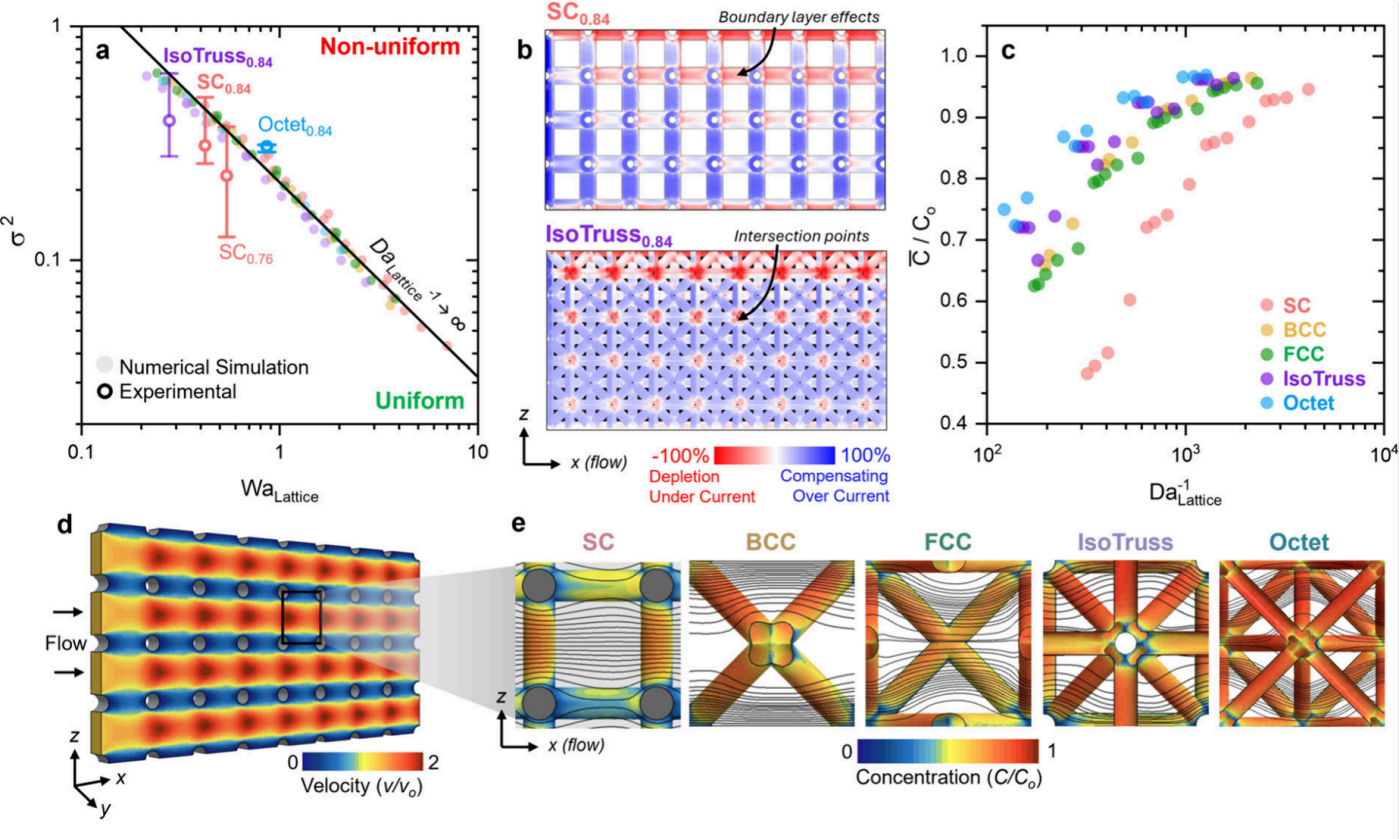
Analysis of global and local nonuniformities as a function dimensionless parameters. (a) Root-mean-square difference (σ^2^) of the current distribution as a function of *Wa*_Lattice_. Experimental data points represent μCT measurements. Numerical simulations were conducted under secondary current distribution. (b) 2D rendering of the IsoTruss_0.84_ and SC_0.84_ samples showing current distribution inhomogeneities. (c) Average electrode surface concentration over the bulk electrolyte concentration as a function of *Da*_Lattice_^–1^. (d) Velocity profile of the SC_0.84_ fluid domain along the *x* axis. (e) Localized surface concentration of each cell architecture.

To further quantify the general macroscopic current uniformity from experimental data and compare to numerical simulations, nonuniformity can be calculated from eq 9 as the normalized root-mean-square deviation:^59^
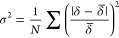
9where δ is the local coating thickness, δ̅ is the average coating thickness, and *N* is the surface number of coating thickness measurements. The numerical predictions of σ^2^ as a function of the *Wa*_Lattice_ dimensionless parameter follow a power law scaling across all investigated structures (Figure 5a). This master curve indicates the utility of *Wa*_Lattice_ for uniformity predictions from secondary current distribution at conditions that are not limited by mass transport (*Da*_Lattice_^–1^ → ∞). However, under conditions where insufficient electrolyte flow is present (corresponding to lower *Da*_Lattice_^–1^ values), mass transport effects become more significant and dependent on the lattice architecture. Hence, using *Wa*_Lattice_ can result in overpredictions of σ^2^, as evidenced by three of the test cases in Figure 5a (except for Octet_0.84_). This is further represented in Figure S4, where insufficient electrolyte flow and concentration effects manifest submicron-level nonuniformities along the *z* dimension of the electrode in the SC and IsoTruss samples.

Reconstructions of the difference between two cell architectures (SC and IsoTruss) of similar porosity are presented in Figure 5b to illustrate periodic occurrences in concentration effects in 3D space. Here, red regions represent a relative decrease in the predicted copper deposition, and blue regions represent a relative increase in deposition necessary to maintain the constant applied current density as a byproduct of concentration effects. The SC lattice develops significant concentration depletion at the top surface. Without significant tortuous mixing, fluid flow is dominated through the center of the unit cells and boundary layers develop on struts that are oriented parallel to the flow direction. Conversely, struts that are perpendicular to flow do not develop such boundary layer effects. The IsoTruss cell type contains six more intersecting beams symmetric about the centroid of the unit cell, including four diagonal struts. Compared to SC, IsoTruss consists of a more homogeneously dispersed density and more strut intersecting variation which leads to more uniform flow field and higher degree of tortuous mixing. The depletion effects are less prevalent in IsoTruss_0.84_ compared to SC_0.84_ except at the topmost unit cells and intersection points. These localized depletion effects at intersections manifest in deviations from the linear relationship with *Wa*_Lattice_ and larger experimental error bars in Figure 5a.

To guide processing conditions, we can determine *Da*_Lattice_^–1^ as a function of the average electrode surface concentration (*C̅*_o_) divided by the bulk concentration of the electrolyte (*C*_o_), shown in Figure 5c. *Da*_Lattice_^–1^ required to achieve uniform concentration distribution *C̅*_o_/*C̅*_o_ ≥ 0.9 for different unit cells is provided in Table 2. In this case, the *Da*_Lattice_^–1^ parameter can be used to guide decisions on the submicron-level uniformity.

**Table 2:** Necessary Da_*Lattice*_^-1^ required to approximate secondary current distribution and validity of Wa_*Lattice*_ approximation

unit cell type	*Da*_Lattice_^–1^ to achieve uniform concentrations, *C̅*_o_/*C*_o_ ≥ 0.9
SC	2280
BCC	800
FCC	920
IsoTruss	600
Octet	440

To further illustrate the effect of flow conditions, *C̅*_o_/*C*_o_ is represented in Figure 5d as an alternative measure of the tortuosity and mixing provided by each cell architecture. From Figure 5e, it is evident that electrolyte aggregates about the center of each unit cell in SC and BCC structures whereas FCC, IsoTruss, and Octet structures are more effective at distributing electrolyte more evenly throughout its volume. It can be inferred that different unit cell types require different *Da*_Lattice_^–1^ to avoid significant concentration effects and be primarily determined by the secondary current distribution regime. As expected, the Octet unit cell provides the most mixing due to the addition of more strut segments as well as having no segments parallel to electrolyte flow. To satisfy *C̅*_o_/*C*_o_ ≥ 0.9, Octet structures require a *Da*_Lattice_^–1^ ≥ 440, meaning that Octet structures can be supplied with 5× less electrolyte flow to equal the same resistance to mass transport relative to activation to that of an equivalent SC structure (*Da*_Lattice_^–1^ ≥ 2280). BCC structures resist concentration effects more effectively as they feature only 45° struts where limited boundary layers can be developed as no strut elements are parallel to the flow direction. These considerations should be made with respect to electrode properties such as mass transport coefficients and pressure drops associated with each different cell architecture.

### 2.4 Mass Transport Engineering Correlations

To evaluate each electrode geometry for mass transport characteristics and comparison to other electrode systems, fluid- and mass-transfer correlations such as the Reynolds, Sherwood, and Peclet numbers can be used to generalize the performance, accounting for differences in porosity, surface area, and beam size. The hydrodynamic behavior of nonuniform channels and architected electrodes is defined by their hydraulic diameter, *d*_h_, or characteristic length, which can be approximated in each structure using eq 10:
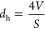
10where *V* is the total wetted volume of the lattice (m^3^) and *S* is the total wetted surface area (m^2^). The Reynolds number (*Re*) characterizes the ratio of inertial forces to viscous forces within fluid flow (eq 11):
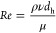
11where ρ is the density of the electrolyte (kg m^–3^) and μ is the viscosity of the fluid (Pa s). The Sherwood number (*Sh*) describes the relative ratio of convective mass transfer to diffusive mass transfer at a surface and can be defined as (eq 12)
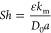
12where ε is the electrode porosity, *k*_m_ is the mass-transfer coefficient (m s^–1^), *D*_o_ is the diffusion coefficient of the electroactive species in the electrolyte (m^2^ s^–1^), and *a* is the internal volumetric surface area of the electrode (m^–1^). The Peclet number (*Pe*) quantifies the relative mass transport of convection to diffusion defined as (eq 13)
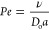
13

**Figure 6:**
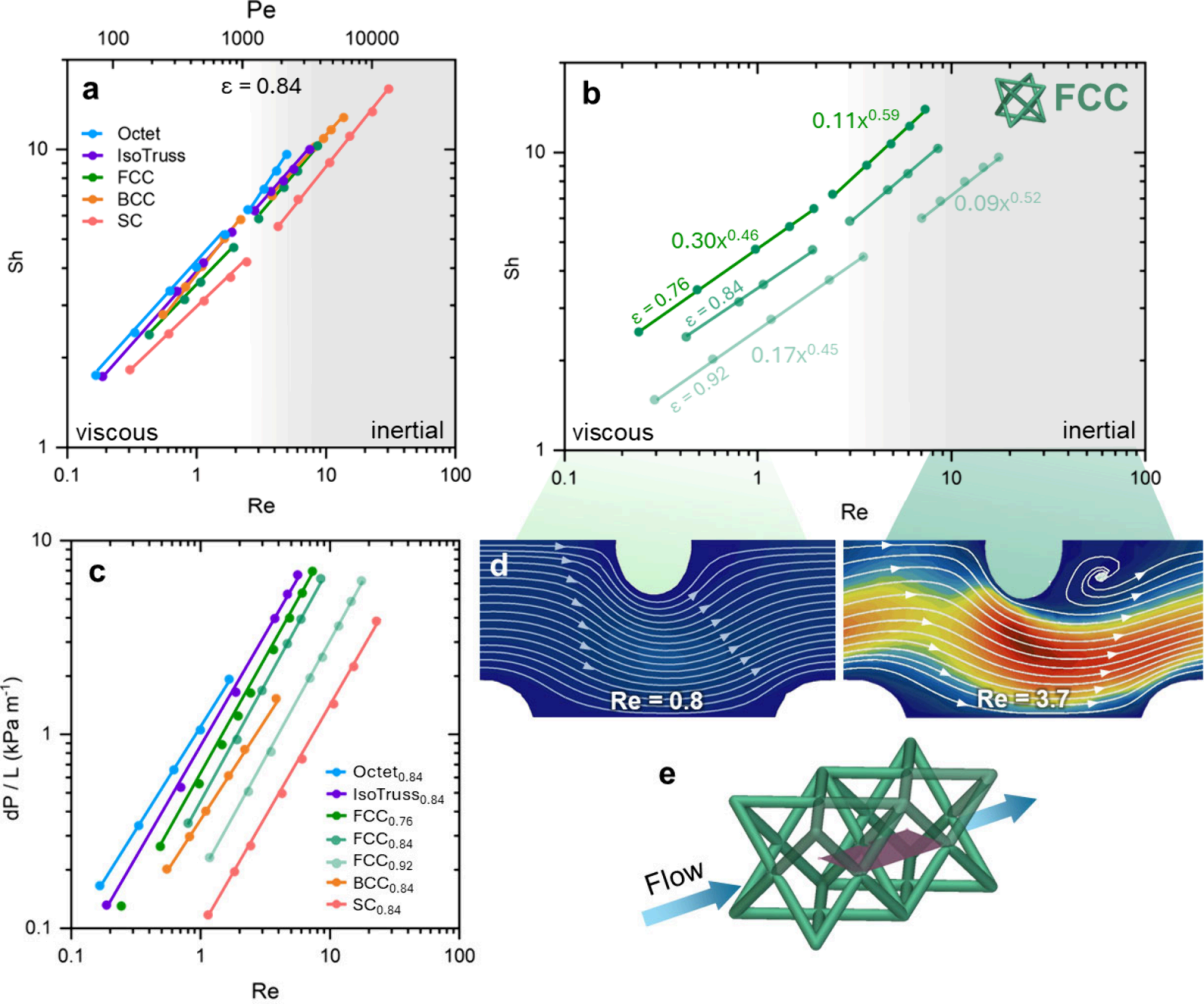
Numerical simulations and analysis of mass transport and flow field effects. (a) Sherwood number represented as a function of the Reynolds and Peclet number from numerical simulations for all unit cell types. (b) FCC-type structures with varying porosity. The gray shading represents the transition from viscous to inertial mass transport regime. Above *Re* > 3, inertial flow effects occur at the electrode surface resulting in increasing power law exponents for more dense structures. (c) Pressure drop vs Reynolds number. (d) Fluid transport through an FCC structure at *Re* = 0.8 and 3.7. At high *Re*, irregular flow tangential to the electrode surface develops increasing the effective mass transfer. (e) Image of the plane location of the fluid transport domain presented (in purple).

To compare to felt, woven, and 3D-printed electrode literature, these architected electrodes can be plotted as the overall mass-transfer coefficient *k*_m_*a* vs *v* derived from the aforementioned numerical model shown in Figure S9. Significant changes in *k*_m_*a* between unit cell types are evident. Mass transport coefficient scales as SC < BCC < FCC < IsoTruss < Octet at low *Re*. Additionally, the types of electrodes were plotted using the engineering correlations in Figure 6a, in which, despite the differences in strut diameters, each electrode can be properly compared. Comparatively, at *Re* = 1, Octet_0.84_ yields a *Sh* 40% greater than that of SC_0.84_. However, a marked trend occurs at *Re* ∼ 3, where fluid dynamics about the architected electrodes transition from inertial to viscous regimes, similarly concluded by Beck et al.^17^

To understand how porosity influences the inertial effects, *Sh* vs *Re* plots are presented at densities ranging from 0.92 to 0.76 in FCC (Figure 6b) structures. Performance and mass transport properties of these electrodes are commonly plotted as a power law relationship and comprised of a prefactor and exponential term. When plotted against *Pe* or *Re*, the magnitude of the exponent indicates the relative effect on mass transport a given increase in fluid velocity has. With a decrease in the porosity from 0.92 to 0.76, the exponential factor in FCC structures in the inertial regime increases from 0.52 to 0.59, whereas in the viscous regime, the exponential factor does not significantly change. Therefore, greater return on mass transport is achieved by increasing flow rate in conditions above *Re* > 1. Conversely, the exponential factor in SC (Figure S10) structure marginally increases with decreases in porosity. This can likely be attributed to the relative number of beams oriented perpendicularly or 45° relative to the direction of flow, where the geometry acts more like a sphere impeding flow as opposed to a flat plate in which boundary layers are dependent on the length of each unit cell.

Cross sections of the velocity profile within an FCC structure (Figure 6d) show that under low flow conditions (*Re* = 0.8) well-behaved fluid patterns develop around the struts. However, as flow increases, transitioning to the inertial mass transport regime (*Re* = 3.7), tangential flow fields develop at strut surfaces due to increased shear stress, as evidenced in the development of recirculation patterns that emerge. The prevalence of such recirculation patterns leads to enhanced local mass transport, which gives rise to higher exponential factors. In denser structures, fluid flow is forced into more tortuous patterns, leading to more frequent and/or high velocity flow instabilities, which increases mass transport but also presents a trade-off in costs associated with larger pressure drops across the electrode. The exponential factor within the inertial regime is significantly different between unit cell types (Table 3). Notably, the BCC unit cell type results in lower exponential factor, which could be attributed to the angle and leading edge of these strut features that cut through fluid flow, resulting in fewer flow instabilities and lower local *Sh* compared to perpendicular strut elements.^60^ Among the unit cells tested, the Octet still benefits from the highest rate of mass transport agnostic comparatively across both fluid regimes.

**Table 3:** Exponential Factor of Mass Transport Correlations for Each Architected Electrode Unit Cell Type

unit cell type	porosity (ε)	viscous mass-transfer exponent	inertial mass-transfer exponent
SC	0.84	0.40	0.53
	0.76	0.42	0.54
BCC	0.84	0.53	0.44
FCC	0.92	0.45	0.52
	0.84	0.45	0.54
	0.76	0.46	0.59
IsoTruss	0.84	0.51	0.45
Octet	0.84	0.47	0.62

Energy and cost of pumping electrolyte through these architected electrodes must be considered in determining the optimal cell structure for a given use application. Relative pumping costs are inferred through numerical analysis of the pressure drop across the simulation domain each different cell type. Improvements to mass transport within these electrodes are tempered by the trade-off in pressure drop across the length of the electrode. Increases in pressure drop mirror that of improvements to *k*_m_*a*. Octet structures have the largest pressure differential, an order of magnitude higher compared to SC structures at similar *Re* (Figure 6c). Decreasing the porosity in architected electrodes increases the pressure drop, as shown for FCC structures. Despite high *Sh* in Octet structures, other electrodes such as BCC may be desired in systems where minimizing the pressure drop is desired.

**Figure 7:**
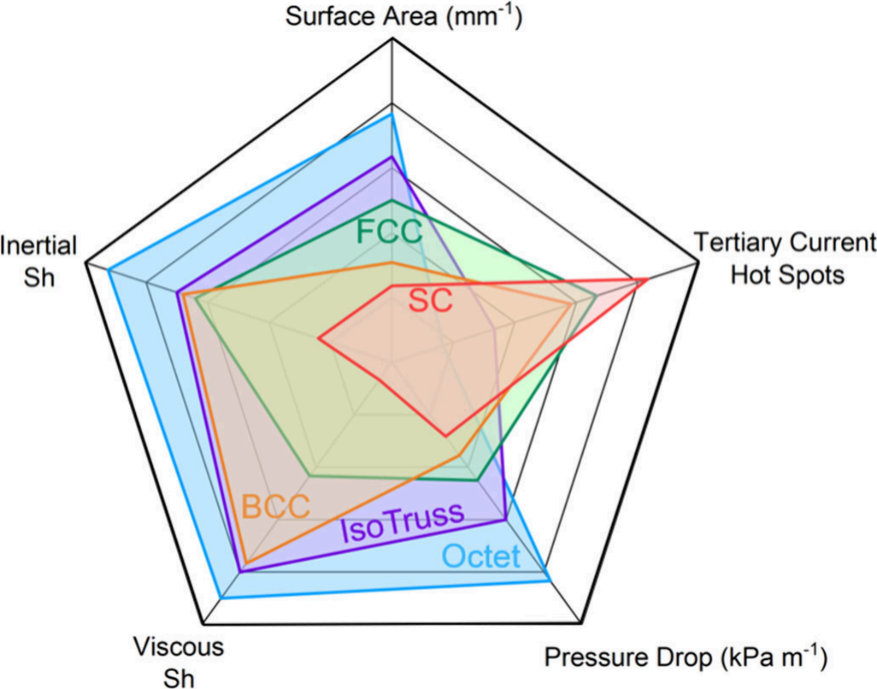
Spider plot of the main fluid and mass transport parameters in this study for different electrode architectures.

The scaling analysis presented here should be used as a guide to determine relative processing conditions over the architected electrodes and be used as a means of systematically studying the growing field of 3D electrode design. Each type of electrode architecture features pros and cons, which are summarized in Figure 7. Octet electrodes are superior in terms of mass transport; however, they suffer pressure drop penalties. BCC electrodes represent a middle ground in mass transport and pressure drop, but also require significantly less electrolyte flow to avoid concentration depletion “hot spots” within a mixed kinetic and concentration dependent regime. Ongoing efforts are focusing on exploring these unit cell geometries for flow battery applications and increasing the effective surface are by reducing the unit cell size and leveraging new additive manufacturing capabilities and detailed resolution.

## 3 Conclusion

This study introduces a platform for evaluating the current uniformity of additively manufactured architected electrodes under electrochemical load. A modified scaling law, *Wa*_Lattice_, was devised to gauge macroscopic current uniformity in architected electrodes based on current density, specific surface area per volume, and the number of unit cells parallel to the electric field. Beam-based lattice electrodes were 3D-printed using SLA, with varied relative densities and unit cell types, then postprocessed into conductive carbon scaffolding. The resulting structures experienced 30% shrinkage but maintained their printed architecture with minimal warpage, exhibiting an electrical conductivity of 500 S m^–1^, determined from in-plane 4-point probe measurements. The carbon morphology can vary depending on the starting material and heat treatment; thus, differences in through-plane conductivity will be explored in feature efforts. In this work, uniform conductivity was used in calculations, and the numerical simulation results were validated using μCT analysis of coating thickness. Modeling efforts determined that *Da*_Lattice_^–1^ can be used as a metric to avoid significant localized submicron level depletion zone and design proper process conditions depending on unit cell types. At sufficiently high *Da*_Lattice_^–1^, *Wa*_Lattice_ can also be effectively used as an accurate measure of the electrodes entire current uniformity, revealing a dependence on beam elements and their angles relative to the flow direction. Overall, mass transport coefficients ranked Octet > IsoTruss > BCC > FCC > SC, with BCC-like structures showing promise for maximizing mass transport efficiency without significant pump losses. These findings hold significant implications for energy storage system design and optimization, offering a pathway toward more efficient and reliable energy conversion technologies.

## 4 Experimental Section/Methods

### 4.1 Printing of Lattice Structures

The lattice structures of varying porosity and architecture were generated in nTop software and printed with PR-48 (Arkema USA), a commercial SLA acrylate resin using a custom microstereolithography printer (LAPμSL) with a 405 nm LED light source.^26^ A light dose of 110 mJ cm^–2^ and layer thickness of 25 μm were used for printing. The green samples were then rinsed and sonicated in isopropyl alcohol to remove uncured resin and then subjected to the heat treatment protocol, including a 1 h hold at 110 °C and a 2 h hold at 190 °C at a 2 °C min^–1^ ramp rate.

### 4.2 Carbonization and Oxygen Plasma Etching

The printed PR48 electrodes were annealed in air at 300 °C for 2 h in a Vulcan furnace under a small ceramic plate to prevent warping, ramping up from room temperature at 2 °C min^–1^. Stepwise pyrolysis process is then conducted in a OTF-1200X 2-in. tube furnace at 250, 400, 600, and 1000 °C with a hold time of 1 h at each step and a heating ramp rate of 2 °C min^–1^ under a N_2_ atmosphere. The annealed PR48 electrodes are then oxygen-plasma-etched in a Harrick Plasma PDC-32G plasma cleaner for 1 h to increase the hydrophilicity of the surface.

### 4.3 Electrodeposition of Carbon Lattice Electrodes

The pretreated carbon electrodes were placed in a custom flow cell for deposition, as pictured in Figure 2a. The electrode is sandwiched between a platinum sheet back contact and Nafion 212 proton exchange membrane to ensure a tight fit. Nafion 212 membranes were treated with 30 wt % H_2_O_2_ for 1 h and then 1 M H_2_SO_4_ at 60 °C for 2 h prior to ensure activation and high ionic conductivity. An additional platinum counter electrode is positioned on the opposite electrolyte chamber and a micro-Ag/AgCl reference electrode is positioned next to the compressed carbon electrode. Silver foil is sandwiched behind each platinum electrode to make electrical contact. An electroplating electrolyte of 0.5 M Na_2_SO_4_ (anhydrous, Alfa Aesar) and 10 mM CuSO_4_ pentahydrate (99.999% trace metal basis, Sigma-Aldrich) (pH = 3) is circulated through both sides of the flow cell, controlled at a flow rate of 36 mL min^–1^ by a using a Masterflex L/S peristaltic pump. Electrodes were deposited at varying prescribed current densities from 0.35 to 1.4 mA cm^–2^ for an average deposit thickness of 27.5 μm. The finished sample was rinsed with deionized water and blow-dried with nitrogen. The sample was dried at 40 °C at 24 h in a vacuum oven and then sealed in a nitrogen-blanketed container.

### 4.4 μCT of Coating Thickness

A Zeiss Xradia 510 Versa X-ray μCT system was used to nondestructively image internal ROIs for four select electroplated electrodes. The ROIs were approximately 5 mm in diameter. Stitching data sets acquired at two vertical positions of the samples allowed the full vertical extent of the samples to be imaged. Imaging smaller horizontal ROIs allowed us to use a higher optical magnification (4×, 3.4 μm pixel size at the detector) objective for the X-ray CT acquisition. The source was operated at a voltage of 130 kV and a current of 77 μA. Based on the observed transmission and per the vendor’s suggestion, the HE2 filter was utilized. The source-to-rotation axis distance was 45.02 mm, while the source-to-detector distance was 60.03 mm; these distances provide geometrical magnification of the sample of *M* = 1.33. A total of 1601 projections were acquired at equally spaced viewing angles along a 190° rotation of the sample. Cone-beam X-ray CT projections are typically acquired over a 360° rotation of the sample, though the small (3.24°) beam angle allows us to achieve similar reconstruction quality with the smaller angular sweep. Each projection comprised a single 15-s exposure, i.e., no frame averaging. Four bright-field projections were acquired at equally spaced intervals throughout the scan. Dynamic ring reduction (DRR) was enabled to reduce ring artifacts due to unresponsive or poorly performing pixels in the detector. DRR applies random lateral (horizontal and vertical) shifts of the sample stage at each projection. The acquired data set was reconstructed using standard filtered back projection on the Zeiss Reconstructor software. Vertical segments within each sample were stitched using the Zeiss Manual Stitcher software. Stitched volumes were then loaded into Volume Graphics VGStudio MAX for analyses. Advanced surface determination was applied to each data set using default settings (search distance of 4 voxels) and from a starting iso-surface chosen visually to be an appropriate threshold to segment out the copper coating. Starting contour healing was set to “Remove particles and all voids”. Coating thickness measurements in 3D were performed based on the determined surface using VGStudio’s “Wall thickness analysis” functionality using the “Ray method” with a search angle of 30° and an expected wall thickness range from 0 to 0.2 mm.

### 4.5 Multiphysics Numerical Modeling

High-resolution, computational fluid dynamic simulations were modeled in StarCCM+ using a half-cell configuration to simulate steady-state local current densities along the architected lattice electrode geometries under laminar fluid flow. Similar numerical simulations were developed for the model CuSO_4_ electrodeposition process.^54^ Relative resistances across the architected electrodes can be inferred by the effective conductive scaling relationship. At the densest structures modeled (ε = 0.76), *k*_m_ = *K*(1 – ε)/σε = 26.1, high enough to assume conductivity in the solid carbon dominates that of the effective ionic conductivity of the electrolyte. For that reason, the electric conductivity of the electrode was assumed infinite for the purposes of the model.

A singular row of unit cells parallel to the direction of fluid flow were modeled assuming the lack of mass transport gradients perpendicular to flow. Incompressible and isotropic flow under the Navier–Stokes governing equation is determined by eqs 14 and 15:
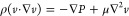
14
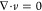
15where ρ is the density of the fluid, *v* is the fluid velocity, *P* is the pressure, and μ is the fluid viscosity. Steady-state kinetics and the transport of electroactive copper species under supporting electrolyte is governed by the Nernst–Planck equation (16):
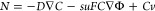
16where *N* denotes the ionic flux, *D* is the diffusion coefficient, *C* is the copper species concentration, *s* is the charge number, *u* is the ionic mobility, *F* is Faraday’s constant, and Φ is the local electrolyte electric potential. Equation 17 is then obtained using the steady-state assumption:
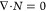
17

The current density *i* is related to the flux of all species and is conserved in the electrolyte (eq 18):
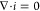
18

Assuming Tafel kinetics at the electrode boundary gives eq 19:
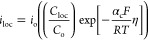
19where *i*_o_ represents the exchange current density, *C*_loc_/*C*_o_ is the dimensionless expression of local concentration relative to the initial concentration of ion species, α_c_ is the cathodic charge-transfer coefficient, *R* is the universal gas constant, *T* is the temperature, and η is the local overpotential at the surface of the electrode. The following electrochemical kinetic parameters were determined from previous efforts determined by rotating disk electrode experiments onto a polished copper electrode:^54^ charge-transfer coefficient α_c_ = 0.485, rate constant *k*_o_ = 9.0 × 10^–5^ m s^–1^, copper species transport *D*_o_ = 3.1 × 10^–10^ m^2^ s^–1^, and electrolyte conductivity κ = 6.4 S m^–1^.

Current distribution of the numerical simulation and coating thickness uniformity determined through μCT analysis is interchanged through the relationship in eq 20:
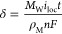
20where δ is the coating thickness, *M*_W_ is the molecular weight of the metal (*M*_W_ = 63.5 g mol^–1^), *i*_loc_ is the local current density, *t* is duration of electrodeposition, ρ_M_ is the density of the metal (ρ_M_ = 8.96 g cm^–3^), *n* is the number of electrons transferred (*n* = 2), and *F* is Faraday’s constant.

## Supporting Information Available

SEM images of carbonized lattices before and after surface treatment, mass-transfer coefficients and Sherwood numbers for different unit cells, and μCT reconstructions and data of copper-coated SC, IsoTruss, and Octet lattices (PDF)
