# Supplementary material for: Understanding the Current Distribution and Mass Transport Properties in 3D-Printed Architected Flow-Through Electrodes
Source: ACS Appl Eng Mater. 2025 Jan 17;3(3):600–12. doi: 10.1021/acsaenm.4c00561 (PMC11960682; doi:10.1021/acsaenm.4c00561)
Supplement: Supplementary file 1 — em4c00561_si_001.pdf [file em4c00561_si_001.pdf]

Supporting Information

Understanding Current Distribution and Mass Transport Properties in 3D-Printed Architected Flow-Through Electrodes

Auston L. Clemens,[1] Kyle Jung,[1] Massimiliano Ferrucci,[2] Megan E. Ellis,[1] Jonathan Tesner Davis,[1] Swetha Chandrasekaran,[2] Zhen Qi,[2] Christine A. Orme,[2] Marcus A. Worsley,[2] Rohan Akolkar,[3] Anna Ivanovskaya,[1] Nikola A. Dudukovic[1,]*

[1]Materials Engineering Division, Engineering Directorate, Lawrence Livermore National Laboratory, Livermore, CA 94550, USA

[2]Materials Science Division, Physical and Life Sciences Directorate, Lawrence Livermore National Laboratory, Livermore, CA 94550, USA

[3]Department of Chemical and Biomolecular Engineering, Case Western Reserve Univeristy, Cleveland, OH 44106, USA

*E-mail: dudukovic1@llnl.gov

Supporting Figures

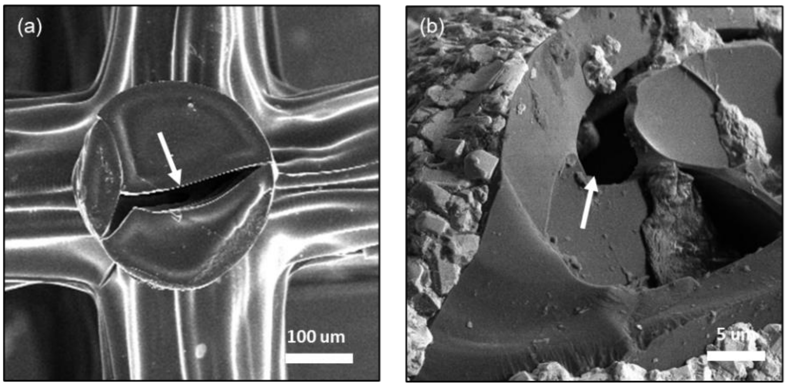

Figure S1. SEM image of gas evolution cracking a) down the center of carbonized $SC_{0.84}$ and b) hollow beam of a broken copper plated beam.

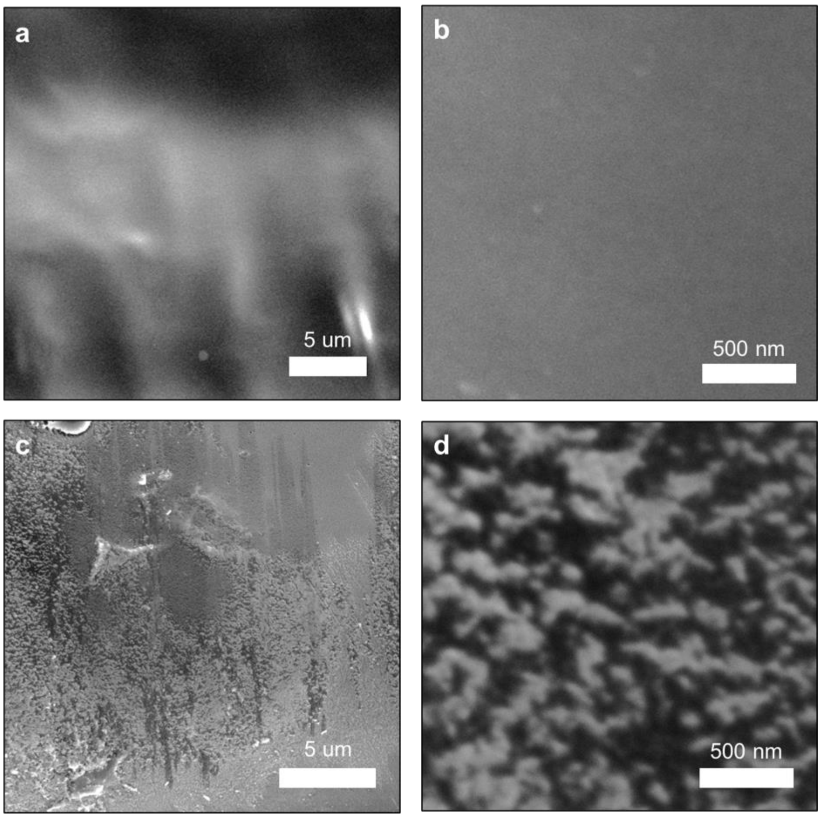

Figure S2. SEM images of carbonized 3D-printed surface before (a-b) and after (c-d) 1 h O_2 plasma treatment. Plasma treated samples yielded rougher surfaces and hydrophilic behavior.

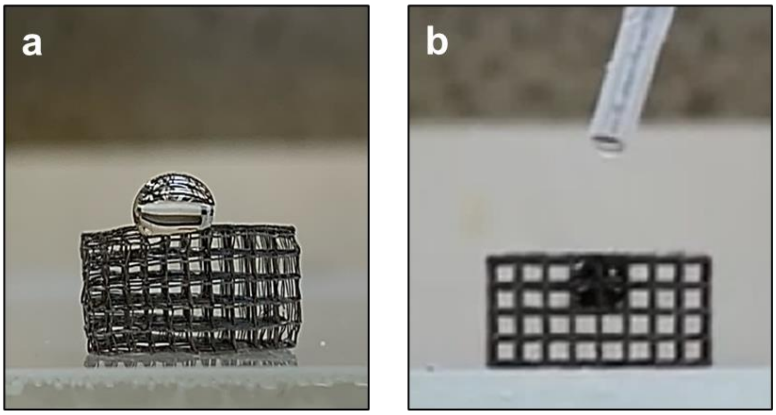

Figure S3. Optical images of a singular droplet of water on a (a) $SC_{0.96}$ without plasma treatment and (b) $SC_{0.76}$ with 1 h plasma treatment.

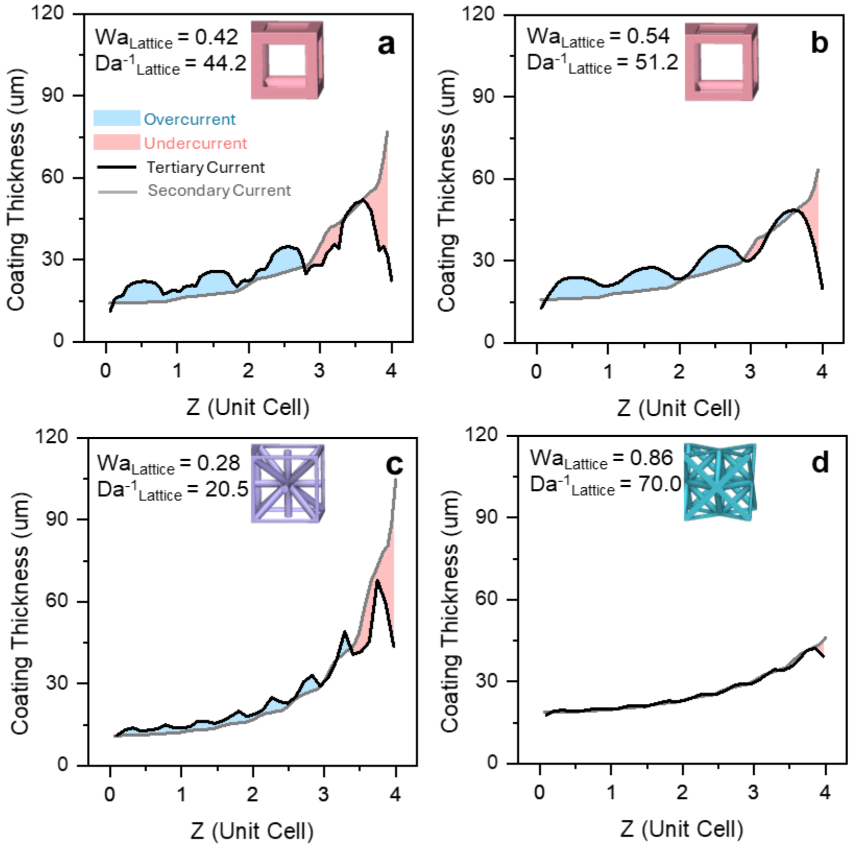

Figure S4. Mass transfer coefficient of 1.75 mm unit cells relative to the average fluid flow rate. (a) $SC_{0.76}$, (b) $SC_{0.84}$, (c) $IsoTruss_{0.84}$, (d) $Octet_{0.84}$.

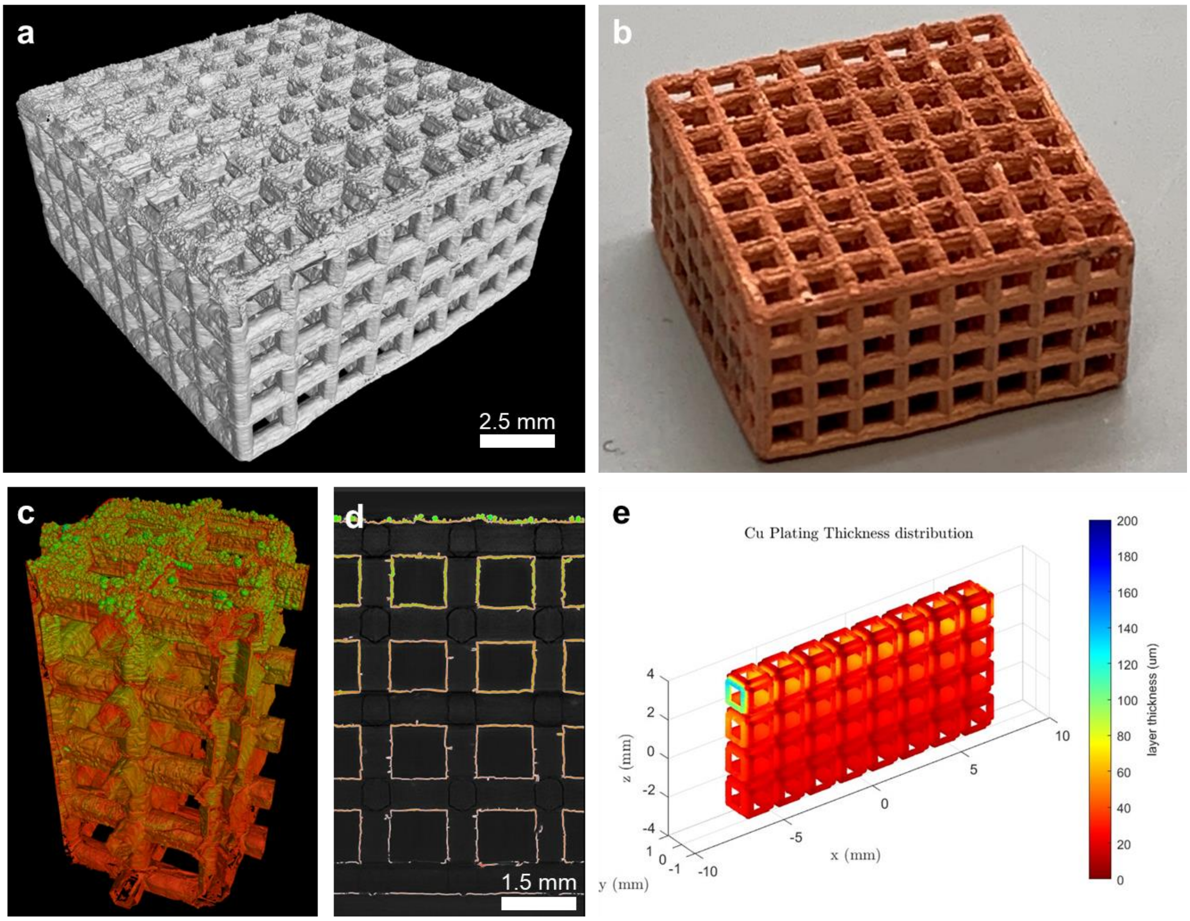

Figure S5. Coating thickness analysis of SC $\varepsilon = 0.84$ depicted in Figure 4d. (a) µCT Reconstruction and (b) image of copper-coated electrode. (c) High resolution region of interest reconstructed sample at the center of the electrode and (d) representative XZ plane of ROI. (e) Modeled 3D point cloud representation of coating thickness for a single XZ row of unit cells.

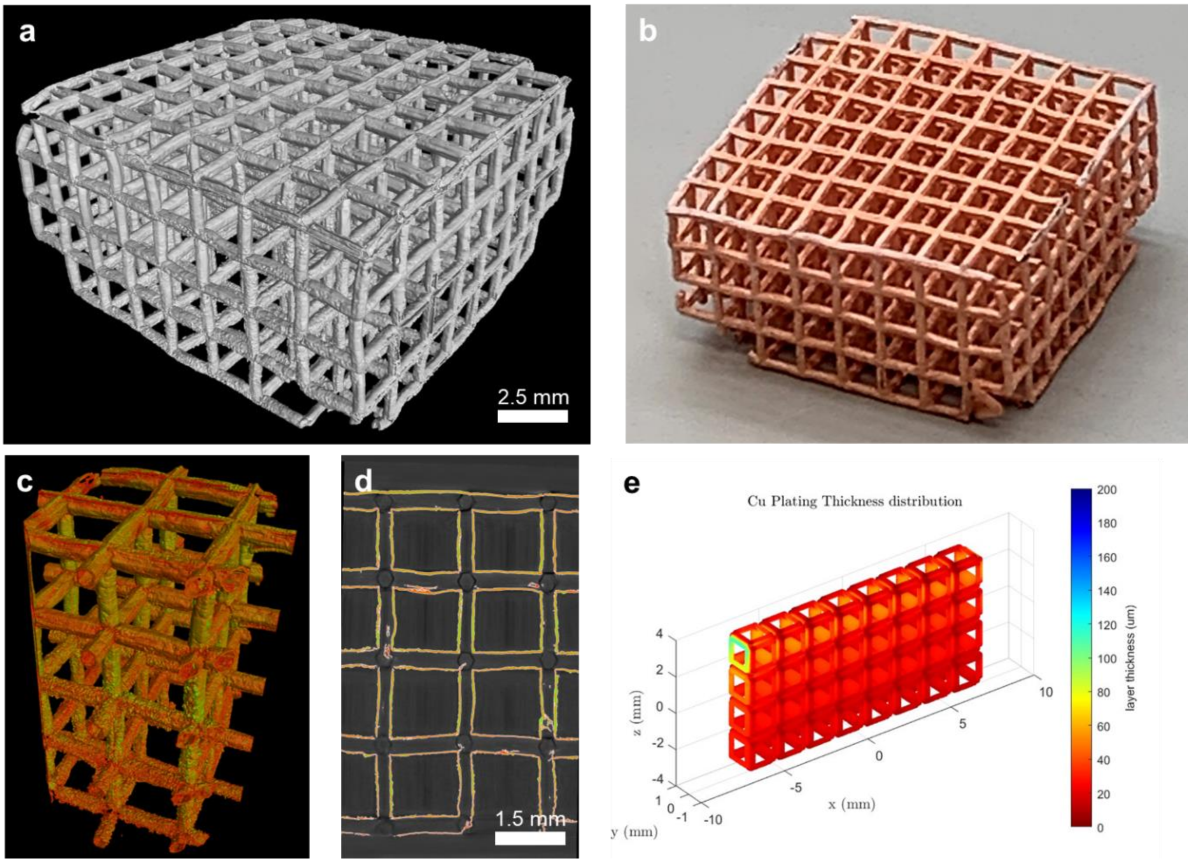

Figure S6. Coating thickness analysis of SC $\varepsilon = 0.76$ depicted in Figure 4d. (a) μCT Reconstruction and (b) image of copper coated electrode. (c) High resolution region of interest reconstructed sample at the center of the electrode and (d) representative XZ plane of ROI. (e) Modeled 3D point cloud representation of coating thickness for a single XZ row of unit cells.

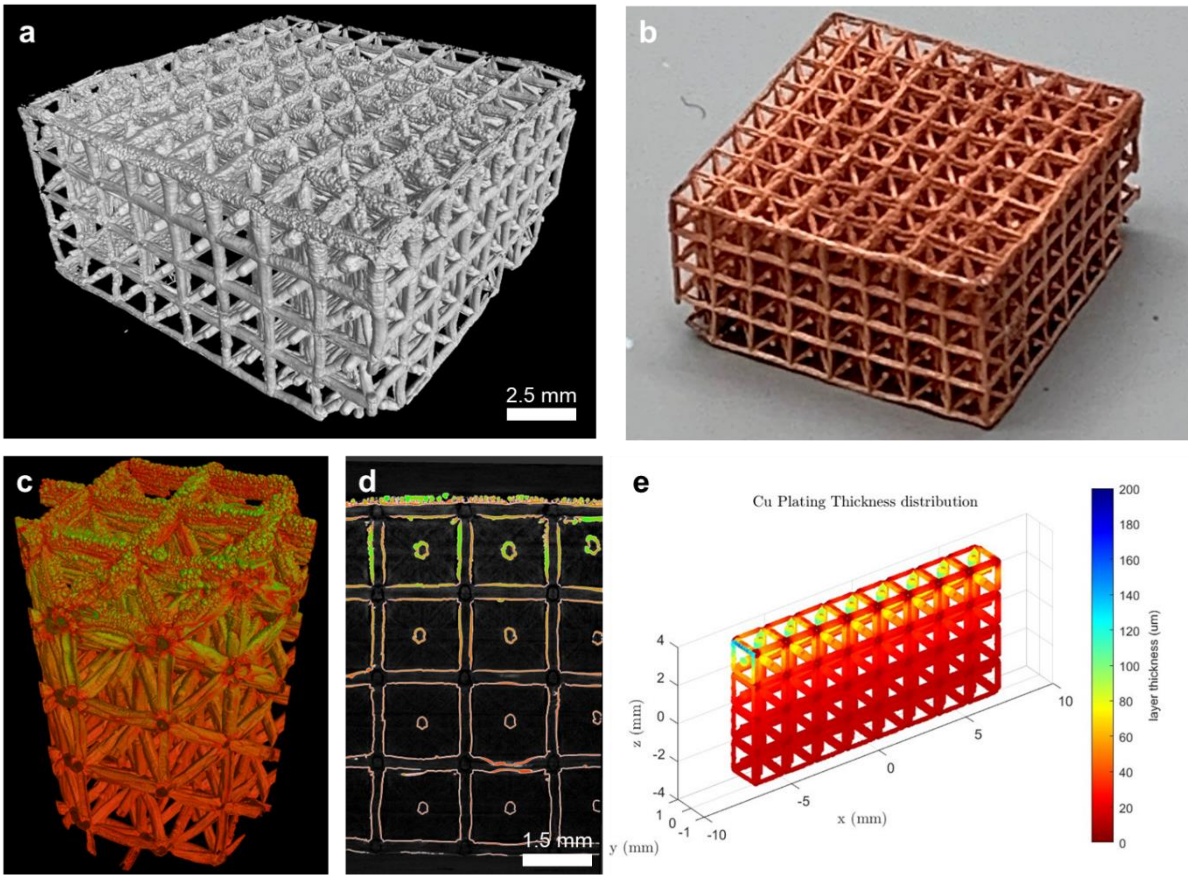

Figure S7. Coating thickness analysis of IsoTruss $\varepsilon = 0.84$ depicted in Figure 4d. (a) μCT Reconstruction and (b) image of copper coated electrode. (c) High resolution region of interest reconstructed sample at the center of the electrode and (d) representative XZ plane of ROI. (e) Modeled 3D point cloud representation of coating thickness for a single XZ row of unit cells.

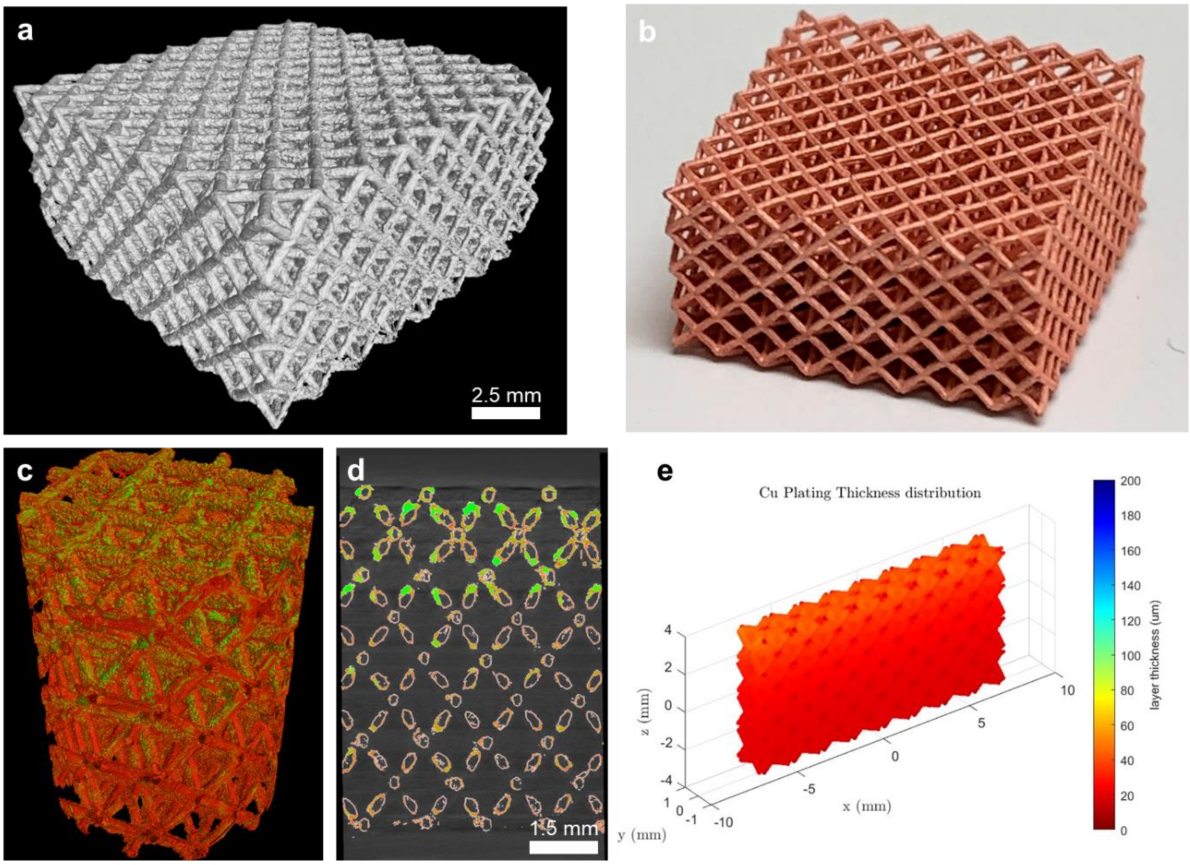

Figure S8. Coating thickness analysis of Octet ε = 0.84 depicted in Figure 4d. (a) µCT Reconstruction and (b) image of copper coated electrode. (c) High resolution region of interest reconstructed sample at the center of the electrode and (d) representative XZ plane of ROI. (e) Modeled 3D point cloud representation of coating thickness for a single XZ row of unit cells.

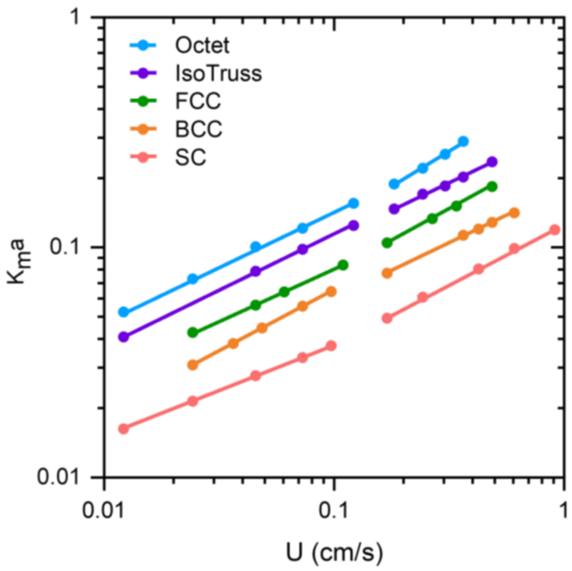

Figure S9. Mass transfer coefficient of each unit cell architecture relative to the average fluid flow rate. Structures were modeled with a porosity, ε = 0.84, and a unit cell size of 1.75 mm.

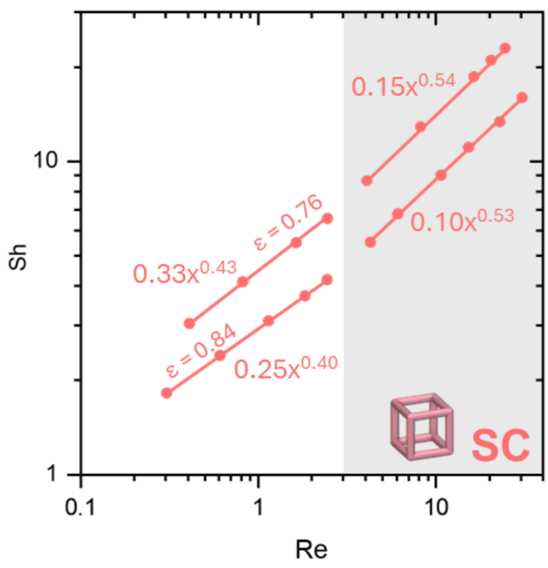

Figure S10. Sherwood Number with respect to Reynolds Number in SC structures with varying porosity. The grey region denotes change from viscous to inertial regime.
